# Alternative work arrangements: Individual, organizational and environmental outcomes

**DOI:** 10.1016/j.heliyon.2023.e21899

**Published:** 2023-11-08

**Authors:** Hasan Yildizhan, Sahand Hosouli, Sıdıka Ece Yılmaz, João Gomes, Chandan Pandey, Tarik Alkharusi

**Affiliations:** a Engineering Faculty, Energy Systems Engineering, Adana Alparslan Türkeş Science and Technology University, 46278, Adana, Turkey; b Faculty of Engineering, Computing and the Environment, Department of Mechanical Engineering, Kingston University London, London, UK; c MG Sustainable Engineering AB, Uppsala, Sweden; d Career Planning Application and Research Center, Adana Alparslan Türkeş Science and Technology University, 46278, Adana, Turkey; e Faculty of Engineering and Sustainable Development, University of Gävle, 801 76, Gävle, Sweden; f Clean Energy Processes (CEP) Laboratory, Department of Chemical Engineering, Imperial College London, London, SW7 2AZ, UK; g Engineering Department, University of Technology and Applied Sciences, Muscat, Oman

**Keywords:** Flexible working, 4-Day workweek, Organizational behavior, Employee behavior, Carbon emission, Environmental impact

## Abstract

Flexible working models are widely used around the world. Furthermore, several countries are currently transitioning to a 4-day workweek. These working models have significant effects on organizational behavior and the environment. The study investigates the employees' attitudes and behaviors toward flexible working and 4-day workweek and the impact on the environment. The semi-structured interview method was used in the study to determine employee attitudes and behaviors; the carbon footprint calculation method was used to determine the environmental impact of a 4-day workweek. According to the study's findings, it has been discovered that there would be a positive impact on socialization, happiness, stress factor, motivation, personal time, mental health, comfort, work-life balance, time-saving, willingness, positive working environment, personal time, and physical health. Furthermore, a 4-day workweek reduced commuting emissions by 20%, resulting in a 6,07 kg tCO_2_e reduction per person. As a result, the study attempted to draw attention holistically to the positive effects of the flexible working model and 4-day workweek. The study is intended to serve as a tool for decision-makers and human resource managers.

## 1 Introduction

An increase in population, current economic developments, and rising living standards in developing nations encourage these nations to use more energy resources [1]. Moreover, the way the business world operates is influenced by current events. Even though many jobs are now performed using various software and automation systems, the human element is still the most crucial and irreplaceable resource in most areas. Recognizing this, many businesses adopt a management approach that aligns with current trends and working styles. These businesses prioritize their employees and strive to understand them. They embody the philosophy of productive organizations that operate harmoniously in this manner [2].

There is a variation in contemporary working styles, which encompasses diverse time schedules. Work schedules can be either full-time or flexible. Additionally, the 4-day workweek model is a contemporary notion that has seen a growing trend in popularity. Employees demonstrate a keen interest in contemporary working styles. For example, employees prefer to work remotely for a variety of reasons, including work flexibility, time savings, convenience, security, and cost savings. While working remotely benefits the individual, it also benefits the environment by reducing electricity consumption and harmful substance emissions [3]. Plus, the adoption of a 4-day workweek, a contemporary approach to work, is increasingly favored in several countries. For example, pilot programs for a 4-day workweek are being tested or implemented in various countries globally. The main reason for this practice is to increase employee satisfaction and, subsequently, productivity. These practices make the organization more appealing and foster a positive recruitment and retention environment [4,5].

According to the literature, quantitative research and questionnaire methods are used to examine the relationship between flexible working and organizational behavior [[6], [7], [8], [9]]. There has not been any research employing the qualitative methodology that comprehensively examines the relationship between organizational behavior and flexible working. Additionally, it is evident that there is a lack of qualitative study in the literature on flexible working [10]. Yet, qualitative studies might enable the acquisition of contemporaneous information and insights into individuals' daily lives and experiences [11]. Moreover, qualitative research has the capacity to offer extensive comprehension and more sophisticated data. This method might offer insightful viewpoints on the topic and present an in-depth examination [12]. By using this approach, we can obtain more precise outcomes. Additionally, flexible working is an issue that affects a variety of academic disciplines. Flexible working overlaps with different disciplines such as environment and economics [13], organizational behavior, and organizational psychology [14]. There are no holistic studies dealing with the subject in the literature. Therefore, studies with a holistic approach are required.

Although the flexible working paradigm is widely adopted in other countries, its prevalence in Turkey is very limited. Moreover, a 4-day workweek has not been executed in the country. It was discovered that only one Turkish business is currently testing the three-month pilot application [15]. Also, there is no scientific study that thoroughly analyzes the 4-day workweek current situation in Turkey in the literature. As a result, understanding the perspectives of Turkish employees on these working models through qualitative studies, identifying environmental impacts, and providing important findings to decision-makers will be extremely beneficial for policy-makers and human resource managers. Furthermore, it is necessary to carry out scientific investigations in nations with diverse cultural attributes to unveil the impact of culture on those activities. Hence, the implementation of comparative scientific research enables the acquisition of more comprehensive insights into the present state of countries with respect to flexible working arrangements.

This study aims to determine the employee attitudes and behaviors towards flexible working and a 4-day workweek, as well as the environmental effects of a 4-day workweek model in a specific company in Turkey. This clarifies the correlation between flexible working models and both organizational behavior and the environment.

## 2 Conceptual framework

Presence at the workplace during the primary time that work is performed, as well as flexibility in the hours of coming to and leaving work outside of this, is referred to as flexible working [16]. The option to choose where, when, and how to work is described as flexibility [17]. Flexible working provides that the employee has the ability to change the timing of her work, such as the start and end times, i.e. she can set the daily or weekly working hours [18].

Over several decades, working practices have changed, creating greater flexibility and mobility. Different working models, like *coworking spaces, digital working hubs, on-demand spaces, and office clubs*, have started to appear in metropolitan areas [13]. The origin of the flexible working model is based on the concept of telecommuting. In the 1970s, Jack Nilles proposed the telecommuting term [19]. Veldhoen developed the activity-based workplace model in the 1990s, which is based on activities rather than employees staying at a single workstation all day [20]. The concept has reached the present day by showing itself in different forms in different ages. The terms used for flexible working overtime have been such as *“co-working spaces,” “on-call employment,” “on-demand work,” “self-employment,” “telework,” “remote work,” “mobile work,” “telecommuting,” and “virtual work”* [21]. Modern technology and the digital age have increased the concept's popularity by allowing for flexible working arrangements [22].

Apart from that, COVID-19 originated from the SARS-COV-2 virus, which was discovered in Wuhan, China [23] and has been one of the most significant events influencing the current way of working. Many individuals used the remote working model, which is a type of flexible working, during the pandemic [24]. For instance, working from home has become more prevalent with the advancement of communication and information technology throughout the pandemic [25]. Numerous positive effects of this working model on both the employee [26] and the environment have been identified during this process [27]. As opposed to that, the COVID-19 epidemic has disrupted the business processes of many companies. It allowed us to see the effectiveness of working from home, which is a type of flexible working during the pandemic period. Many businesses managed and succeeded during this time period by having employees work from home [28]. Due to the pandemic, quarantines were enforced, which made it difficult for enterprises to conduct their regular operations. Because businesses are unable to operate from their typical central location, numerous individuals have begun working from home [24]. Mayer & Boston conducted a survey with 794 employees in New Zealand and showed that they were quite satisfied with working from home during the pandemic period, and 82.6% of the participants wanted to switch to part-time or full-time work from home after the pandemic [24].

The lockdown imposed in response to the pandemic had both positive and negative consequences. During these times, traffic on the way to work had decreased significantly and had even come to a halt. It had reduced the resulting air pollution and carbon emissions in this case. As a result of the pandemic, the business world now has a new hybrid working model. Employees were given the option to work remotely under this new model. This model's most significant contribution is its positive effects on environmental health [29]. Furthermore, these lockdowns have provided numerous economic, social, and environmental benefits by eliminating the need to commute to and from work or by shortening the time and distance to the co-working area. As a result, it is emphasized that by drawing attention to the sustainability of the new working model in studies, this model will significantly contribute to the solution of energy and environmental problems [30]. These contributions are not limited to energy and environment. Therefore, it is necessary to examine the effects in detail.

This study employs the Ability-Motivation-Opportunity (AMO) theory as a framework to assess the impact of flexible working arrangements on organizational behavior and environmental effects. According to Appelbaum, Bailey, Berg & Kalleberg, the idea posits that human resource management (HRM) strategies that enhance a business's human capital by improving human capabilities can lead to positive performance outcomes, including enhanced productivity, decreased waste, improved quality, and higher profitability [31]. Flexible working has the potential to enhance the abilities, motivation, and opportunities of employees. The adoption of flexible working arrangements often requires employees to adapt to new technologies and communication tools. By participating in training programs and actively honing their skills, employees can bolster their capacity to operate effectively in a remote or flexible work setting. Flexible working arrangements, such as the option to work remotely or to choose flexible hours, can serve as significant motivators for employees. Providing flexible work arrangements that align with employees' preferences and lifestyles might correlate with higher job satisfaction levels, thereby boosting motivation and fostering improved behavioral outcomes [[32], [33], [34]]. Moreover, executing clear and well-supported policies on flexible working arrangements encourages employees to utilize these options, which in turn promotes positive organizational behavior. As a result, improvements in AMO components may enhance employee productivity [35,36]. Evidence suggests that HR procedures play a pivotal role in shaping employees' attitudes and overall performance.

Additionally, incorporating Stakeholder Theory, which underscores the importance of addressing the needs and aspirations of employees, further strengthens the theoretical foundation of the research. This theory delves into the relationships between organizations and their stakeholders – groups or individuals who can either influence or be influenced by the activities and decisions of an organization. Theoretical stances argue that companies should not solely focus on maximizing shareholder value but must also consider and cater to the interests of all stakeholders [37,38]. Employees are typically considered major stakeholders within an organization. This theory stresses the significance of individual interests, needs, and behaviors. Flexible working arrangements are often seen as a strategic measure to meet the needs of employees seeking a better work-life balance and reduced commuting-related stress [39]. Furthermore, the theory emphasizes the crucial role stakeholders play in achieving sustainable performance, which encompasses both social and environmental aspects. By meticulously evaluating stakeholder interests and adopting ethical, long-term perspectives, organizations can champion sustainability, fostering a responsible business environment. Such an approach not only benefits society and the environment but also augments the enduring success of the organization [40].

Turkish businesses implement HRM strategies that are based on American models. The adoption of Western-based HRM practices in Turkey became increasingly prevalent following the 1990s [41]. According to Tüzüner Turkey's transition from personnel management to HRM was driven by the American model. Small and medium-sized organizations (SMEs) and large-sized enterprises (LSEs) have different HR arrangements. Turkish human resources incorporate Turkish cultural norms in SMEs, but some LSEs have embraced or are adopting a Western-style HRM organization and procedures. Also, foreign-capitalized enterprises in Turkey have Western-style HRM practices [42]. Additionally, the prevalence of digital human resources systems in Turkey has been noted to have increased significantly since 2020 [43]. Based on the literature, it can be asserted that the utilization of both Traditional Human Resources Systems and New Human Resources Systems is prevalent in contemporary Turkey. The system employed is subject to variation depending on the specific enterprises.

### 2.1 The impact of flexible working

Flexible working has a variety of impacts. Flexible work arrangements can have an impact on an individual, an organization, and the environment. This study focuses on what kind of effects it will have on employee attitudes and behaviors on an organizational basis. It also focuses on the environmental consequences.

#### 2.1.1 Organizational impact

##### 2.1.1.1 Positive organizational impact

This working model is recognized to have a variety of effects on the employee. For instance, flexible working improves employee performance [26,36,44] and employee engagement [45]. A 6-month study of 64 federal employees revealed that flexible working increases employee satisfaction [46]. Research indicates that flexible, shared workplaces affect job satisfaction [34] and organizational commitment [47]. A more flexible work schedule increases organizational commitment [8].

This working model has positive effects on organizations as well. For example, the findings of a four-month experiment with 246 employees in five production units of a financial institution revealed that flexible working for two units increased productivity [48]. Likewise, a recent study discovered that working remotely increases productivity [49]. According to Van der Voordt’s analysis of the costs and benefits of flexible workspaces, cost savings were not just attributable to direct expenses like reducing space but also to indirect benefits like increased employee productivity and shorter commuting times [35]. This demonstrates how flexible working can cut costs significantly and produce savings for businesses while also promoting environmental protection.

Flexible working increases employment appeal, which benefits firms in terms of both recruiting and retention. It was found that perceived flexibility fit was positively associated with job-seeking and job-acceptance intentions [50]. According to a study that used semi-structured interviews with 108 home care professionals, flexible working improved facility operations and had a positive impact on both staff and management. Flexible working arrangements can support HRM policies and practices by promoting recruitment and retention [51].

##### 2.1.1.2 Negative organizational impact

Flexible working negatively affects turnover intention, which is an important organizational result that can be seen frequently among employees and can negatively affect the organization [52]. When employees have more control over their work schedules and flexibility, turnover is reported to be lower [53]. Additionally, virtual communication is extensively utilized in the context of flexible working. Yet, a study states that virtual communication reduces team cohesion [54].

#### 2.1.2 Individual impact

##### 2.1.2.1 Positive individual impact

One of the most significant benefits of this working model for the employee is the existence of physical space. Physical space is important for employee motivation. Using flexible working, the individual could somewhat alter the physical work environment. Flexible working has a significant impact on employee motivation [33]. A study showed that employees in a crowded and busy work center feel exhausted and tired before their work starts [55]. The study revealed that a person's motivation levels would be higher if they worked from home or in other predetermined workplaces.

Flexible working hours contribute to and facilitate work-life balance [39]. It has been identified as a highly effective strategy for enhancing employee well-being since it enables employees to effectively manage their duties outside of work [56].

##### 2.1.2.2 Negative individual impact

Adjusting working hours allows a person to strike a balance between work and life. Flexible working hours have been shown to reduce work-family conflict [57]. As a result of this balance, the level of stress experienced can be reduced [39]. The practice of remote work has been found to have a detrimental impact on individuals' social interactions. This scenario poses challenges for persons whose social inclinations hold prominence. According to Büssing's study, participants expressed a sense of longing for interpersonal communication and the opportunity to share their accomplishments with others [58]. Also, a study examining the disadvantages of remote working showed that distance employees face communication problems [59].

#### 2.1.3 Environmental impact

##### 2.1.3.1 Positive environmental impact

For the sake of the environment, it is imperative to reduce total energy use. The energy output of the fuel and the resultant Carbon dioxide (CO_2_) emissions are cumulative. Diesel also has the potential for high cumulative energy consumption [60]. Moreover, the greenhouse gas emissions from burning fossil fuels, resulting in carbon dioxide, have the most significant environmental impact of all those negatively affecting the earth [61]. The high carbon footprints that emerge significantly hamper future efforts to achieve low carbon levels [62]. Using a flexible working model, employees can either work from home or another location. This setup can alter the environmental impacts, affecting energy consumption both at home and during transportation [63]. In a study with 100 employees in a university department in Cyprus, it was revealed that an hour of remote work saves 4000 L of transportation fuel and 7400 kg of carbon dioxide using ArcGIS, Life Cycle Analysis, and fuel consumption analysis methods [30]. Another research analyzed commuting data from employees across twenty-four countries, using twelve transportation modes, to determine the greenhouse gas emission reductions achieved by remote work. This study spanned two years and included 815 participants, covering both pre-COVID-19 and COVID-19 eras. Remote work reduced commuting emissions by 43% in 2019 and 97% in 2020, leading to a reduction of 1.9 tCO_2_e per individual [27]. A travel and work habits survey of 514 individuals, both users and non-users of Remote Work Hubs (RWHs) in Dublin's city center, aimed to identify potential emissions and travel time savings. The results showed RWH users traveled, on average, 60 km less daily, with most arriving at work later. 34% drove to their regular workplaces, while 12% commuted to RWHs. Based on these data, solo drivers could reduce CO_2_ emissions by 1126 tons annually by working at an RWH three days weekly. The findings underscored significant travel time and emissions savings through RWH utilization [64].

Ge, Polhill, and Craig explored various workplace-sharing programs, studying the correlation between co-working and travel habits. Their results indicate that adopting workplace sharing in expansive organizations can notably decrease traffic and transportation-related air pollution, especially CO_2_ emissions [65]. This research accentuates the environmental benefits of workplace-sharing initiatives. Studies show that flexible work schedules help decrease emissions of harmful gases, thus fostering a healthier environment [66]. A comparison of the CO_2_ footprints of home office practices in Germany, the UK, Sweden, Italy, Spain, and the Czech Republic, conducted by the Vodafone Institute, revealed that a more adaptable work model might reduce Germany's CO_2_ by 12.2 million tons [67]. Conversely, flexible work is seen to cause less environmental degradation, yielding more positive outcomes. A study based on impact analysis indicates that a realistic “two-day-a-week” nationwide remote work strategy in Ireland could significantly reduce annual car commuter miles by roughly 1 billion. This has associated societal benefits, such as decreased emissions and personal time savings [68]. Moreover, such a practice bolsters sustainability by lessening individual carbon emissions [69].

##### 2.1.3.2 Negative environmental impact

The use of flexible working arrangements results in reduced spatial requirements for organizations, leading to cost savings in terms of running energy and utilities [69].

Although there is no single and best method to minimize the impact on the environment, the right combination of working from home and working from the office on different days depending on job roles is critical. This combination is achieved by deciding on an effective working method by considering factors such as type of organization, type of work, location of employees and organization, commuting distances, travel modes for commuting.

The implementation of flexible working has an impact on the level of individuals, organizations, and the environment. Table 1 was created using the above-mentioned studies from the literature.

**Table 1:** The classification of the effects of flexible working.

Organizational	Individual	Environmental
Organizational productivity (+)	Time-saving (+)	Reduction in carbon emissions (+)
Recruitment and retention (+)	Motivation (+)	Sustainability (+)
Turnover (−)	Work-family conflict (−)	Energy use (−)
Employee performance (+)	Work-life balance (+)	
Employee commitment (+)	Well-being (+) Stress (−)	
Employee satisfaction (+) Team cohesion (−)	A feeling of isolation (−) Communication (−)	

### 2.2 4-Day workweek

The 4-day workweek is not a novel concept. Ever since the 1970s, the 4-day workweek has sparked interest and debate. Since then, there have been various trial applications. It is viewed as one of the alternative work arrangements like flexible working. Flexible working and 4-day workweek are not the same thing. Contrary to flexible working, a 4-day workweek is regarded as a compressed workweek because of decreasing working days per week. People believe that a 4/40 workweek comprised of a 4-day workweek and a 3-day weekend from Friday. However, it is indicated that any mixture is feasible under the 4/40 framework [70]. A 5-day work week consists of 8-h days and is known as 5/40. A 4-day workweek is defined as 4/32 [71]. One of the most common compressed schedules is also seen as a 10-h day, or 4-day week [32].

There are numerous pilot implementations and real-world implementation studies underway in various countries today. For instance, the federal government of Belgium passed the “New Labor Agreement” in June 2022. Employees' working hours, salaries, and social rights are unaffected by this regulation. Employees have the option of working four days instead of five. They are able to work 9.5 h per day, a total of 38 h per week, and take three days off under the arrangement, which aims to provide peace, comfort, and time for themselves outside of work. The goal of this agreement is to transition to a more innovative, sustainable, and digital economy. It is expected that employee satisfaction will increase productivity. Researchers discovered that worker stress and burnout decreased, and that life-work balance improved. Furthermore, the four-day study is being piloted in various countries and companies. In recent months, a “4 days a week” trial was conducted in the United Kingdom, and 86% of the participating companies stated that they would maintain the 4-day working policy. Furthermore, there is a global significant interest in the 4 working day model. Some pilot applications have been conducted in companies in the United States, Canada, Australia, and New Zealand. Spain will begin a trial period. Iceland has already established itself as a forerunner in the 4-day workweek. A government trial for a 4-day workweek is set to begin in 2023 in Scotland. Wales is indeed contemplating a trial [72].

The option “In favor of 4-day week, 10-h day with Monday or Friday as the day off” was endorsed by 68% of participants in research with 1744 employees regarding a 4-day workweek, while 32% disapproved. Another option, “In favor of a 4-day week with days off during the week,” received approval from 24% of respondents but not from 76%. Those who objected cited fatigue, conflict with evening activities, problems with child activities, conflict with family activities, and too boring as their reasons [73]. Research compiled and analyzed the compressed workweek studies. The results show that attitudes toward the compressed week are optimistic. Organizational attitudes and behaviors such as job satisfaction, home and personal life, leisure and recreation, and absenteeism are generally positively affected; however, fatigue has been found to be negatively affected. Despite the fact that no study found a decrease, performance outcomes are vague [32]. These investigations were conducted as the idea first started to take hold in the 1970s, It would therefore be more beneficial to take into account recent studies and contrast their findings.

According to a study on alternative compressed work weeks as part of a 4-day week, employees working the 4/10 workweek have lower levels of work-family conflict than other employees who work the normal work schedule. Additionally, employee productivity and ability to serve citizens were increased. However, there was no statistically significant difference in most measures of job satisfaction [74]. A qualitative analysis method was used in a study that focused on the 4-day workweek and the remote work of agile software development teams. According to the study, these two working models increased job satisfaction and productivity. However, the team members' stress levels increased. The study also confirmed the effects of team members' reduced social interaction [75]. A qualitative analysis method was used to conduct research in a company that manufactures car parts and uses a 4-day workweek in England. According to the findings, participants reported increased productivity and a significant decrease in turnover rate. Furthermore, participants emphasized that the organization has become a more appealing place to work. Other benefits include increased recruitment and retention, reduced stress, more time with family, and more engaged employees [5]. The benefits of a 4-day workweek have been shown as recruitment and retention, productivity, well-being, less stress, cost saving, focusing more on developing skills, qualified work, fewer sick, and happiness [4].

Studies have also shown that working 4 days a week saves money and conserves energy. In research in Florida, all schools in the area were closed every Friday from June 7 to August 10, 2009, in order to save money on services. The majority of participants expressed satisfaction with the program. The utilization was reduced by 11.8 million kWh, which saved $900,000 in costs. Moreover, $156,585 in fuel expenses were saved. The program's overall fuel and electricity savings came to $1,056,585 [76].

While the 4-day workweek is being implemented and various trials are being conducted around the world, the question of how the situation is in Turkey arises. A hybrid working model has begun to be used with the pandemic in Turkey. Although some companies continue to use the hybrid model after the pandemic restrictions have expired, there is currently only one company in Turkey that uses the 4-day workweek. Aksa Akrilik has tried the 4 days a week working practice in a corporate setting for the first time in Turkey. It has employed 200 people and works 4 days a week from January 1st to March 31st, 2023. Furthermore, the company continues to work from home two days per week. The application's stated goal is to increase productivity and employee satisfaction [15].

Working Hours and Model Study in Turkey, which included 241 companies and 1130 young people, revealed that young people prefer to work 4 days per week. The 4-day workweek is in high demand among company human resources managers and young people [77]. However, no such initiative has yet been launched.

As a result, first and foremost, a suitable environment might be established for the 4-day workweek by increasing flexible working. Although the 4-day workweek is not currently being implemented in Turkey except for Aksa Akrilik, different flexible working models can be expanded. Remote working or hybrid models, for example, should first become more common. Sure, these working models can have a wide range of effects on various variables. As a result, the research findings will serve as an important guide.

The study's research questions that are inferred from the existing literature are as follows:

RQ1 What are the impacts of flexible working or a 4-day workweek on organizational behaviors?

RQ2 What is the perspective of the employees on the implementation of a 4-day workweek?

RQ3 What are the environmental impacts associated with the implementation of a 4-day workweek?

## 3 Materials and methods

The analyses utilized in the study were conducted inside the boundaries of the same organization. The organization functions within the industrial sector with a primary emphasis on the manufacturing of steel. The organization predominantly employs individuals in blue-collar workers, while also having a contingent of white-collar employees. The corporation engages in international trade by exporting its products to several countries. The employee profile exhibits a national framework.

### 3.1 Carbon emission calculation

In this study, three different types of buses were examined based on their fuel consumption, average age, passenger capacity, and average CO_2_ emissions. Among these types, the M-BUS stands out as the most efficient option, consuming only 6.2 L of diesel per 100 km and emitting an average of 155.5 g of CO_2_ per kilometer. In comparison, the MID-BUS consumes more fuel at 8.3 L per 100 km and has a higher CO_2_ output of 217.1 g per kilometer. The third type, OT-BUS, exhibits the highest fuel consumption, requiring 20 L of diesel per 100 km, and emits the most CO_2_, with an average of 800.1 g per kilometer. Additionally, the M-BUS, MID-BUS, and OT-BUS have average passenger capacities of 18, 24, and 46, respectively.

In assessing the carbon footprint of diesel-fueled buses, a standardized methodology was applied. The CO₂ emissions (g/km) of each bus type were calculated using the relation: CO₂ emissions (g/km) = Fuel Consumption (l/100 km) × Emission Factor [78]. Here, the emission factor corresponds to the CO₂ produced per liter of diesel fuel combusted, and is established at 2680 g of CO₂ per liter [[79], [80], [81]]. This value is derived from the inherent carbon content of diesel and the understanding that the combustion of 1 g of carbon yields approximately 3.67 g of CO₂. The selected method and associated emission factor align with recommendations from prominent global institutions such as the Intergovernmental Panel on Climate Change (IPCC). Adopting this standardized approach facilitates an accurate and comparative assessment of CO₂ emissions across distinct bus categories.

**Fig. 1:**
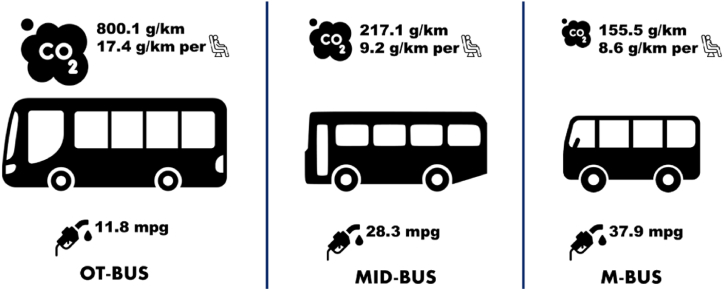
Comparison of bus types: average CO_2_ emissions per passenger.

Based on these findings, it is evident that the M-BUS offers the best combination of fuel efficiency, CO_2_ emissions, and passenger capacity among the three types of buses based on average CO_2_ emissions per passenger (see Fig. 1).

In order to assess the environmental implications, this study examines a specific scenario where 1428 individuals commute to their workplaces. The transportation for this commute is provided by a total of 68 diesel buses and minibusses, which are categorized into three types: 39 M-BUS, 24 MID-BUS, and 5 OT-BUS. The study evaluates the fuel consumption and CO_2_ emissions considering both 4-day and 5-day work weeks. The calculation formulas used to determine the overall fuel consumption and CO_2_ emissions take into account the quantity of each bus type, their respective fuel consumption rates, and the average levels of CO_2_ emissions.

The monthly total distance traveled has been computed by utilizing available data for each work commute route, which takes into consideration factors like the average number of trips per month and the round trip distance measured in kilometers. Furthermore, the monthly fuel consumption in liters has been estimated for each route, taking into account the specific type of vehicle used (M-BUS, MID-BUS, and OT-BUS). A similar approach has been employed to calculate the monthly total CO_2_ emissions. The emissions for the 4-Day Plan have been calculated for each route using the monthly total CO2 emissions and taking into account 18 working days per month. Similarly, the emissions for the 5-Day Plan utilize the same calculated monthly total CO_2_ emissions but consider 22 working days per month.

### 3.2 The effect of flexible working and 4-day workweek on employee organizational behavior

One of the study's objectives is to understand the impact of flexible working and a 4-day workweek on employee organizational behavior. For this reason, the study employed a qualitative method. Qualitative methods are employed to address inquiries pertaining to the experience, meaning, and viewpoint of participants [82]. Semi-structured interviews are a widely used technique within the scope of the qualitative method. Semi-structured interviews were employed due to their suitability for examining attitudes, opinions, and feelings, which are inherently challenging to directly observe [83].

#### 3.2.1 Setting and study area

White-collar employees were determined for the qualitative research. The present circumstances can be attributed to the unsuitability of blue-collar workers for flexible work arrangements, resulting in potential disruptions to production. The decision has been made to select white-collar employees whose working conditions are conducive to flexible work arrangements. The data were collected from the white-collar staff of the steel manufacturing company. The study employed maximum diversity sampling as the chosen sample method. The company employs 120 white-collar employees who work in departments including sales and marketing, information systems, foreign trade, logistics, quality control and quality assurance, financial affairs, planning, project and investment, purchasing, strategy and business development, administrative affairs, internal audit research and development, occupational health and safety, and human resources. Data were gathered from all departments for the purpose of this study. The objective is to gather data encompassing a diverse array of perspectives pertaining to the research topic [84].

In qualitative investigations, it has been observed that any subsequent data collected after a certain point replicates the data already presented by the sample in the earlier stages of the research [85]. Therefore, interviews were carried out until the point of data saturation was reached, resulting in a sample size of 35 employees.

#### 3.2.2 Data collection tools

Semi-structured interviews are distinguished by the utilization of open-ended inquiries and the employment of an interview guide that outlines the general topics of interest [86]. Therefore, the study employed the semi-structured interview methodology as a qualitative research method to facilitate employees' articulation of their thoughts and views, drawing from their individual experiences. It aims to expose the actual thoughts of employees regarding flexible working and a 4-day workweek.

During the initial phase of formulating the semi-structured interview form, the first and third authors did a comprehensive assessment of relevant literature regarding the research topic. Subsequently, they utilized the information gathered from the literature to develop the semi-structured interview form [39,49,51,57].

The perspectives of three professionals were sought on the interview form, including an academician with expertise in qualitative research, a specialist in human resources, and a human resources manager. Based on the data derived from expert comments, the researchers conducted a thorough reassessment of the interview form, focusing on the attributes of clarity, suitability, and adequacy of the questions. Subsequently, appropriate revisions were implemented. The interview questions are shown in Table 2.

**Table 2:** The interview questions of the study.

No.	The interview questions
1	What do you think are the advantages of flexible working and a 4-day workweek for individuals?
2	What advantages do flexible working and a 4-day workweek bring to the company?
3	Will the 4-day workweek be successful in Turkey? Please explain with reason.
4	Which employee/organizational attitudes and behaviors do you think will enhance most with flexible working or a 4-day workweek? (Organizational commitment, organizational belonging, willingness to stay at work, employee performance, employee satisfaction, employee productivity, work-life balance, organizational communication)
5	If you think that flexible working or a 4-day workweek will not be effective on employee attitudes and behaviors, please explain the reason.
6	If you have anything to add about flexible working and 4-day workweeks, please specify.

Employees were informed about the subject of the meeting and interviews were performed at the company where the employees worked during business hours by making an appointment in advance. The interviews were conducted face-to-face in a quiet environment. Interviews of a duration ranging from 20 to 25 min were held with employees who provided consent for voice recording during the interviews, and their voices were fully recorded. The recorded interviews were then transcribed and analyzed one-on-one.

#### 3.2.3 Validity and reliability

Validity refers to the comprehensive evaluative assessment of the extent to which theoretical justifications and empirical data substantiate the sufficiency and suitability of interpretations [87]. Guba & Lincoln developed four distinct criteria, namely credibility, dependability, confirmability, and transferability, with the aim of enhancing the validity of qualitative research [88]. Firstly, prolonged involvement was employed in order to enhance the credibility of the findings. Then, member verification was conducted by reviewing the responses to verify their accuracy. In conclusion, a peer debriefing session was undertaken with one academic researcher specializing in qualitative investigations, as well as two experts in the field of human resources [89]. For dependability, investigator triangulation was carried out with the involvement of more than one investigator during the collection, analysis, and interpretation of the data [90]. Also, the study presented the objectives, purpose, study process, methods and procedures employed, interview questions, significant statements, categories and themes, and study findings in terms of confirmability [89]. Lastly, transferability encompasses the extent to which the findings of a study can be applied to other contexts or samples [88]. However, qualitative research is not designed to achieve generalizability. All experiences are centered around comprehending the unique circumstances of the individual. In order to enhance the transferability of the study, a comprehensive discussion was conducted on the experiences of the participants, and illustrative examples were used. Consequently, scholars examining the research can utilize the findings in their respective endeavors. Furthermore, the methodology employed in selecting the sample for transferability was thoroughly described, along with a comprehensive explanation of the participants' characteristics and the interview context [91].

Reliability describes the likelihood that consistent outcomes will be obtained across various researchers and participant cohorts when employing identical measurements within a particular study. It signifies the extent to which research findings can be consistently reproduced [92]. The participants were instructed to prioritize honesty in order to ensure the accuracy of the collected data. Hence, the interview was carried out in a conversational manner. Moreover, the participants were provided with comprehensive details regarding the research's objectives and methodology, as well as the utilization and preservation of the data acquired from them. Besides, with regard to the aspect of reliability, an experienced academic specializing in qualitative research was assessed collaboratively during the analysis phase [93]. Finally, ensuring data integrity through the utilization of a recording device, presenting the empirical results devoid of subjective analysis, and engaging in a comprehensive discussion of the findings within the conclusion section are other precautions implemented to enhance the reliability of the study. Along with these, the study materials and methodology were subjected to ethical committee authorization at Adana Alparslan Türkeş Science and Technology University (06/2).

#### 3.2.4 Data analysis

Thematic analysis—one of the qualitative analytical techniques—was employed for data analysis. In a data set, such as a collection of interviews, focus groups, or a collection of texts, the thematic analysis describes the procedure of seeking recurring patterns of meaning. This analysis method consists of the steps of getting acquainted with the data, creating preliminary codes, searching for themes, analyzing themes, defining and labeling themes, and producing reports [94].

The study was executed using MAXQDA software. Initially, the accuracy of the transcriptions was verified by reviewing them with the original records. Subsequently, the transcribed text was imported into the MAXQDA program. Each participant was given a label from P1 to P35. The coding and categorization were conducted by the first and third authors, employing methodological approaches. The process of open coding was employed to assign codes to various ideas, while categories were afterward formed based on the distinct traits and dimensions of these ideas [95]. The initial step involved the coding of qualitative data that exhibited similarities and interconnections. Subsequently, comparable codes were incorporated inside the same categorization, establishing thematic groups. These processes were afterward validated by the research team through discussion.

In addition to this, word cloud analysis was used to visualize the content in the research. The word cloud is one of the most popular ways to graphically display the data of a text [96]. Words that are related to each other in a text are determined according to their frequency ratios, and the text is represented visually [97]. *Wordart* program was used for word cloud analysis in the study.

## 4 Results

### 4.1 Carbon footprint results

According to Fig. 1, the data illustrates the commuting patterns of 1428 individuals to their workplaces in this study. The primary mode of transportation for the majority of the routes is the MID-BUS, followed by the M-BUS. In total, there were 1430 trips completed in a month using the M-BUS, 1536 trips using the MID-BUS, and a significantly lower number of 192 trips using the OT-BUS.

**Fig. 2:**
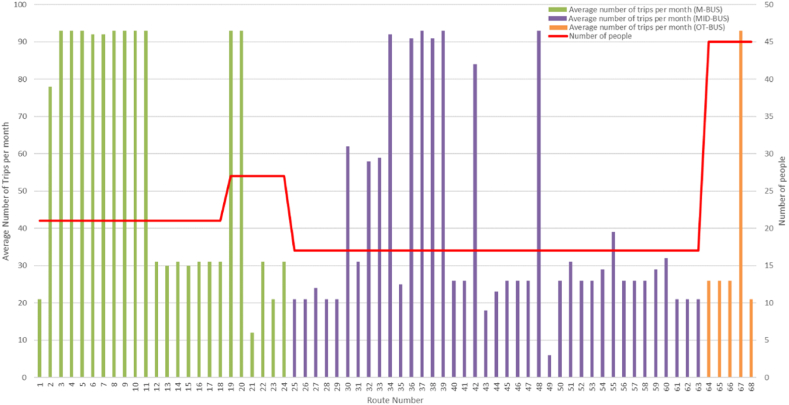
Commuting patterns and trip counts by bus type.

**Fig. 3:**
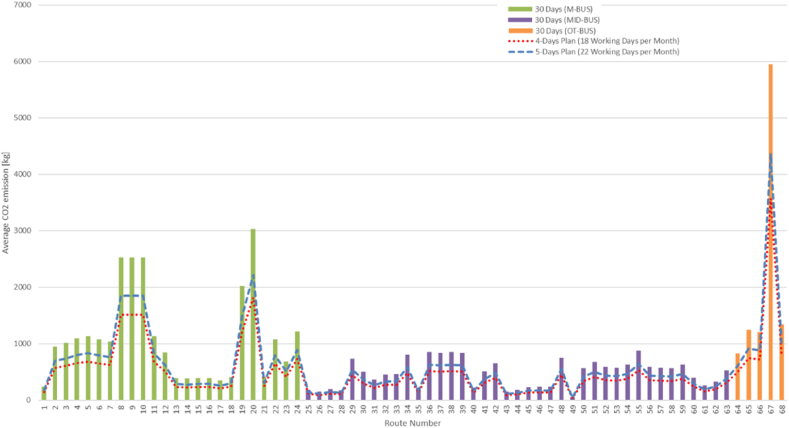
Average CO_2_ emissions comparison: 4-day and 5-day plans vs. 30-day months.

Fig. 2 presents valuable insights into the potential for reducing overall CO_2_ emissions by implementing more efficient transportation plans. By comparing the emissions for the 4-day and 5-day plans to the emissions for a full 30-day month, we can assess the impact of reducing the number of operating days (see Fig. 3). Both the 4-day and 5-day plans yield substantial reductions in average CO_2_ emissions when compared to a full 30-day month. The 4-day plan demonstrates an average reduction of approximately 40.4%, while the 5-day plan achieves an average reduction of about 27.2%. Such reductions highlight the potential for improving environmental sustainability and aligning transportation practices with global climate change mitigation goals.

### 4.2 Qualitative analysis results

The study was carried out to find out the employee attitudes and behaviors regarding flexible working and a 4-day workweek with a qualitative analysis method. 35 white-collar employees in total—25 men and 10 women—were interviewed. 71.4% (25 persons) of the participants are between the ages of 18–30, 20% (7 persons) between the ages of 31–43, and 8.57% (3 persons) of them between the ages of 44–56.71.4% (25 persons) of the participants have a bachelor's degree and 28.57% (10 persons) have a master's degree.

**Fig. 4:**
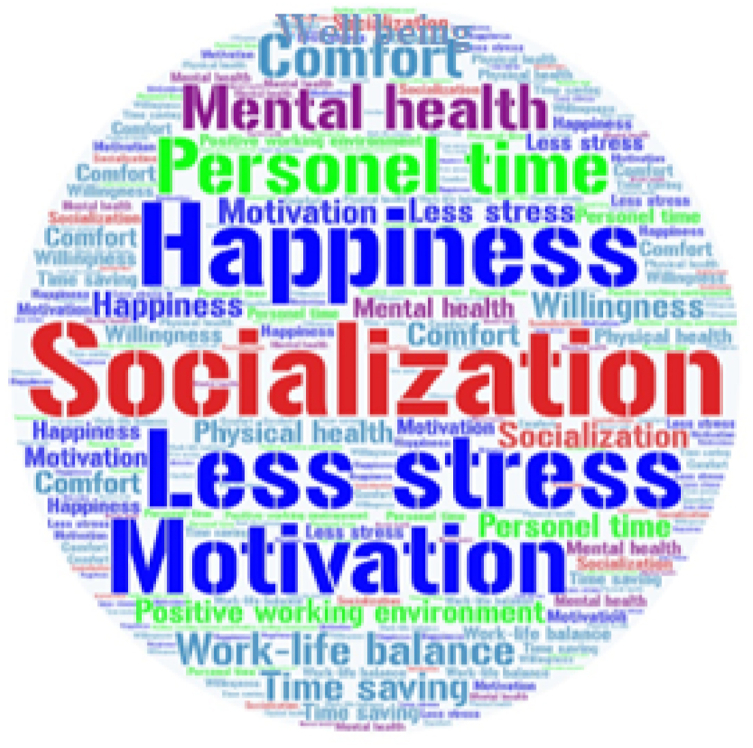
Word cloud analysis results.

Firstly, word cloud analysis was carried out through the *Wordart* program to determine the advantages of flexible working and a 4-day workweek for individuals. The primary basis for the visualization was the frequency of recurrence of the words used in this research. The word cloud analysis results regarding the advantages of flexible working and a 4-day workweek for individuals are shown in Fig. 4. The participants described the advantages of flexible working and a 4-day workweek with the words that are socialization, happiness, less stress, motivation, personal time, mental health, comfort, work-life balance, time-saving, willingness, positive working environment, well-being, personal time, and physical health.

All the answers of the participants were reviewed considering thematic analysis. Appropriate categories were created after reviewing the results. The two themes were determined through discussions with three experts and the research team. Following this stage, the relevant categories were added to the themes. Table 3 displays the categories and themes of the questions asked about the advantages that flexible working brings to the company. Organizational productivity, organizational performance, employee productivity, and employee performance are the top three categories among the increasing factors while organizational costs, employee costs, and job errors are the top three categories among the decreasing factors.

**Table 3:** The increasing and decreasing factors affected by flexible working and 4-day workweek.

Increasing factors	Frequency	Decreasing factors	Frequency
Organizational productivity	20	Organizational costs	16
Organizational performance	15	Employee costs	10
Employee productivity	10	Job errors	5
Employee performance	10	Turnover rate	3
Organizational commitment	8	Occupational accidents	2
Employee satisfaction	7		
Positive organizational environment	5		
Organizational belonging	5		
Recruitment	3		
Retention	3		
Production	3		
Time-saving	2		
Organizational efficiency	1		
Customer satisfaction	1		
Team cooperation	1		
Organizational growth	1		
Organizational power	1		
Employee-employer relationship	1		

**Fig. 5:**
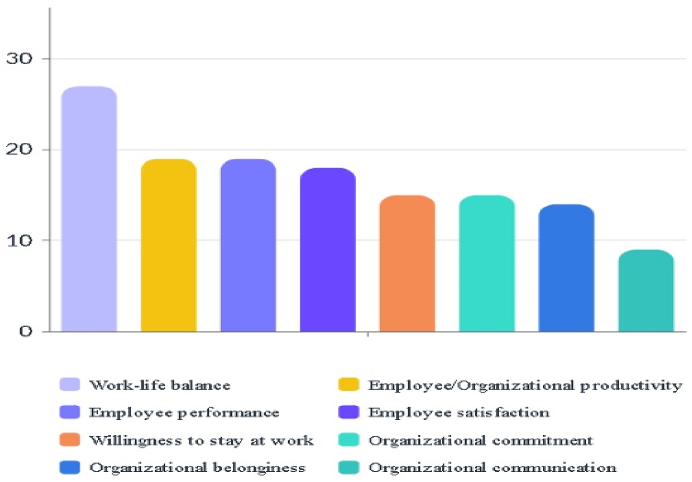
The bar chart result.

According to the findings, the bar chart was created to show which employee/organizational attitudes and behaviors increase the most among organizational commitment, organizational belonging, willingness to stay at work, employee performance, employee satisfaction, employee productivity, work-life balance, organizational communication with flexible working or 4-day workweek. It is shown in Fig. 5. According to the results, work-life balance, employee/organizational productivity, and employee performance are respectively the most affected top 3 concepts.

**Fig. 6:**
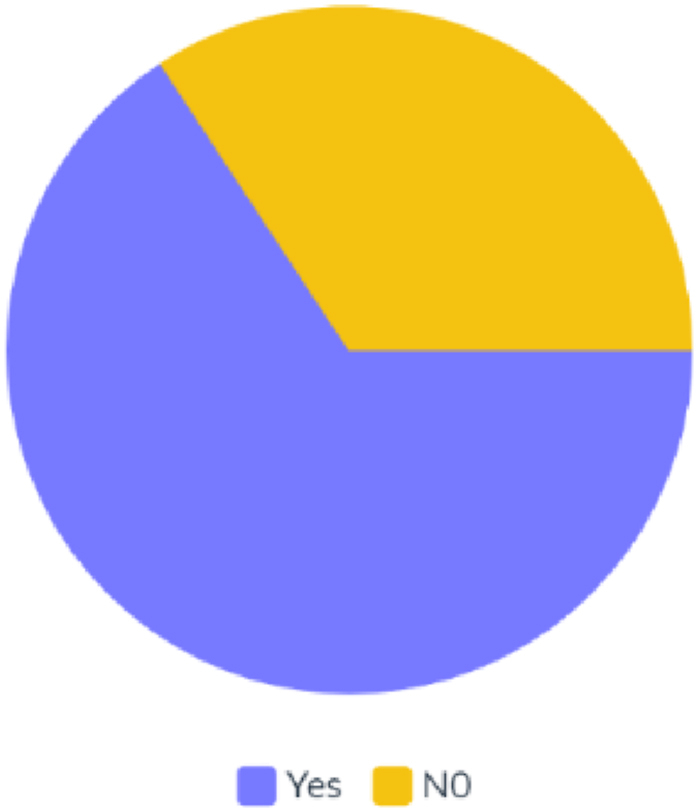
The results of flexible working or a 4-day workweek about being successful.

The majority of participants believe that flexible working or a 4-day workweek will be successful in Turkey when asked their opinions on the subject. The results are shown in Fig. 6, most participants think that it will be successful with a rate of 65.71%, and others believe that it will be unsuccessful with a rate of 34.29%.

Table 4 displays the participants' responses who had a gloomy outlook. These remarks indicate that, in terms of employee attitudes and behaviors, some participants believe that organizational commitment and organizational belonging will be positively affected by the results in Table 3 and Fig. 5, while others believe that they will be negatively affected. This study is not adequate to establish a clear relationship between flexible working or a 4-day workweek and organizational commitment and organizational belonging. It is clear that further research is required to ascertain the effects on organizational commitment and belonging. It is acknowledged that the majority of those who think negatively are pessimistic. The impact of the current economic climate situation is what led to this circumstance. As a result, it is anticipated that economic issues will cause it to collapse. There is also concern that it will lead to uncertainty and a change in the status quo. The cultural factors proposed by Hofstede also lend credence to this conclusion. This is consistent with Hofstede's assessment of Turkey's high degree of uncertainty. Uncertain circumstances are not preferred in Turkish culture. People try to avoid ambiguity [98].

**Table 4:** Participants’ negative responses to the success situation of flexible working or a 4-day workweek in Turkey.

Code	Participant Responses
*P5*	*“It can be abused by employees. The concepts of work discipline and work-life balance should be well understood by employees. In addition, employers may apply to mobbing and increase the workload as it will make the employee work less. In other words, employers also can abuse this situation.”*
*P7*	*“It creates uncertainty, I don't think it will be successful. It may be successful in business lines where workforce and standardization are sufficient. However, it cannot be successful in manufacturing. To be successful in manufacturing, the workforce capacity must be sufficient. Scope should be determined, analyses should be made for business lines, it should be investigated whether there is sufficient workforce capacity for these business lines. If not, can workforce adequacy be provided with education, this situation should also be examined.”*
*P20*	*“Such a thing is not possible in Turkey. We are a civilization that grows as we produce; if we stop producing, the economy will become out of balance. In our country, where hot money is returned, daily money is needed, and if we do not work even for a day, we will be adversely affected by this situation.”*
*P31*	*“Leaving the workplace can lower commitment to the job and the organization; employee engagement may decline as a result of alienation from the organization, it may have a negative impact on organizational* belonging*, and collaboration amongst employees may decline. It is necessary to research both its economic and social implications.”*

Table 5 displays the participants' responses who had a positive outlook. As may be inferred from the remarks, it is believed that this working model will enhance productivity, allow employees to spend more time with their families and friends, and have a beneficial impact on the work. The statement that salaries shouldn't decrease was made. Also, it has been stated that expenses may drop significantly.

**Table 5:** Participants’ positive responses to the success situation of flexible working or a 4-day workweek in Turkey.

Code	Participant Responses
*P4*	*“Turkey needs to test out this system. It will be successful in a diligent and pragmatic society like Turkey if it is successful in working societies like Japan.”*
*P9*	*“Yeah, it directly affects time management; productivity rises as the person spends more time with his family and himself; it is simpler to gain efficiency from those who have a social life; they are more upbeat and produce high-quality work. Yet, salaries shouldn't go down.”*
*P18*	*“Certainly, I believe it will be more appropriate, particularly for the younger generation. The workforce and potential will grow as a result. Because of the company's increased efficiency, costs will be cut indirectly.”*
*P35*	*“Undoubtedly, due to the rapid pace of labor, occupational accidents are rising in Turkey. Hence, workplace accidents can be avoided. Positive effects are shown on both the employees' physical and mental well-being. However, there should be absolutely no reduction in salaries.”*

## 5 Discussion

Firstly, an examination was carried out on the transportation data pertaining to a group of 1428 employees utilizing diesel buses and minibusses. By analyzing the travel patterns and associated emissions of this specific group, insights were gained regarding the potential impact of implementing a 4-day workweek model on the transportation sector and the resulting environmental implications.

The round trip distance covered amounted to 6976 km. Over the course of a month (30 days or 7 days a week), a total of 25135.8 L of fuel was consumed, with an average of 837.9 L per day. The total distance traveled within the month reached 314,443 km, averaging around 10481.4 km per day. Consequently, the total CO_2_ emissions for the 30-day period were calculated to be 65029.92 kg, resulting in an average of 2167.7 kg emitted per day.

To compare the emissions between a 4-day plan (18 working days in a month) and a 5-day plan (22 working days), the study found that the 4-day plan emitted 39017.9 kg of CO_2_, while the 5-day plan emitted 47688.6 kg. This indicates a difference of 8670.6 kg between the two plans (20% reduction).

These findings emphasize the significant impact that reducing working days can have on CO_2_ emissions. Implementing a 4-day workweek could result in notable environmental benefits by reducing carbon emissions. These results can serve as valuable insights for decision-makers and organizations interested in adopting more sustainable and flexible working models, considering the positive environmental impact and potential reduction in fuel consumption. By prioritizing work-life balance, reducing commuting trips, and minimizing carbon emissions, organizations can contribute to a more sustainable and employee-friendly work environment.

This section of the research paper focuses on the findings pertaining to the implementation of a 4-day workweek. The results of the qualitative analysis indicate that employee/organizational productivity and employee/organizational performance are favorably impacted by this 4-day workweek. This result is supported by studies in the literature [4,26,49]. Also, it was discovered that the implementation of a 4-day workweek would result in lower organizational costs, employee costs, and job errors. The findings regarding the cost are consistent with the literature [35]. On the other hand, the participants' views on flexible working and a 4-day workweek were not entirely apparent. It was observed that the majority continues to think it would succeed. Nevertheless, there remains a prevailing sense of pessimism regarding the implementation of a 4-day workweek. The primary factor contributing to this disease is potentially associated with the existing economic circumstances of the country. Another factor is that, as was already indicated, Turkish culture may have relatively low tolerance levels for ambiguity. However, the results indicate that employees see a potentially positive impact on their attitudes and behaviors (for example, having personal time, socialization, work-life balance, increasing motivation, less stress, etc.), suggesting that a 4-day workweek could be advantageous. In particular, socialization, happiness, less stress, and motivation concepts have been identified as significant advantages. This finding is similar to some studies in the literature [4,5,33,39].

Consequently, the ambiguity around the 4-day workweek can be mitigated through the establishment of comprehensive frameworks and the implementation of more pilot projects. Furthermore, the study yielded findings indicating that employees exhibited a lack of consistency in their understanding and interpretation of the constructs of organizational commitment and belonging. These variables may be subject to further investigation in future research endeavors.

## 6 Conclusion

This study investigated the attitudes and behaviors of employees toward flexible working, a 4-day workweek, and the environmental effects of a 4-day workweek model at a specific Turkish business. Despite the widespread adoption of the flexible working model in many nations, it has not reached the desired level in Turkey. Additionally, even though the 4-day workweek has begun to be officially implemented in many nations, this study aims to portray the current perspective of the workforce before this working model is implemented in the future; this concern should also be addressed in Turkey. The implementation of such practices, as observed in other nations, should also be explored in Turkey. Employee attitudes and behaviors in the workplace are likely to be positively influenced because of this practice. It would be advantageous to introduce this practice by conducting pilot applications in various businesses and business lines and observing the results. Furthermore, salaries should not be reduced in these practices; working hours should not be increased daily, and workloads and working hours should be aligned with an effective model.

This study aims to expand the existing body of research by offering novel perspectives and a deeper understanding of the relationship among flexible working arrangements, organizational behavior, and effects on the environment. The research conducted in this study established theoretical frameworks that consider the intricate relationship between human behavior, group dynamics, and organizational systems within the context of flexible working arrangements and environmental sustainability. Furthermore, the study makes a valuable contribution to the existing body of knowledge on flexible working by introducing a novel research methodology. The existing body of literature on flexible working is found to lack a comprehensive discussion on qualitative research. Hence, the present study reveals significant findings derived from the utilization of semi-structured interviews, a qualitative research technique, in the context of the flexible working literature.

This study specifically addresses flexible working or a 4-day workweek with a holistic approach. The study incorporates a theoretical contribution through the integration of multiple disciplines. The research integrated various fields including organizational behavior, human resources, work psychology, and environmental sustainability, thus enabling a more holistic understanding of the subject.

Practical contributions can be categorized into three main classifications: organizational practices, policy recommendations, and sustainability initiatives. This study has the potential to provide valuable insights to businesses regarding the advantages associated with the adoption of flexible working arrangements, such as flexible working or a 4-day workweek. The study is significant for human resource managers contemplating adopting reduced work schedules, considering a 4-day workweek. The study's emphasis on the advantages of flexible working may serve as a catalyst for businesses to implement policies that promote work-life balance, employee autonomy, and job satisfaction. Consequently, this might strengthen the overall well-being and productivity of employees. It also aims to shed light on how these practices can have a positive influence on employee behavior and contribute to environmental sustainability. The results of this study have the potential to offer empirically supported perspectives for policymakers and governmental organizations in their efforts to formulate or revise policies pertaining to flexible work arrangements and environmental sustainability. Additionally, the study is designed to facilitate the advancement of environmentally sustainable practices within businesses, including the mitigation of carbon emissions associated with commuting, reduction of resource utilization, and minimization of waste production.

### 6.1 Future studies

The results are a guide and offer crucial ideas for future studies. Longitudinal studies can be carried out in future studies. This enables researchers to track the impact of flexible working practices on both organizational behavior and environmental consequences over a prolonged duration. This methodology has the potential to offer valuable perspectives on the enduring viability of flexible work arrangements and their effects on employee well-being and environmental performance. Conducting a comparative analysis of businesses that exhibit different degrees of adoption and execution of flexible working practices may facilitate the identification of key aspects that lead to favorable outcomes. A potential avenue for scholarly inquiry is examining the impact of corporate culture, leadership styles, industry features, and geographical settings on the relationship between flexible working arrangements, organizational behavior, and environmental outcomes. Developing an investigation into the cultural factors that impact the level of acceptance and efficacy of flexible work arrangements within diverse cultural settings might yield significant insights. Comparative studies can be conducted to investigate the impact of cultural norms, beliefs, and expectations on employee attitudes, behaviors, and the environmental effects related to flexible working. A broad understanding of the implications and challenges associated with flexible working and its effects on organizational behavior and the environment can be attained by taking into account the perspectives of several stakeholders, including managers, policymakers, and environmental advocacy groups.

### 6.2 Limitations

One of the drawbacks inherent in this study is the lack of generalizability of the findings, as they were derived only from a qualitative research approach [91]. Additionally, the study may exhibit evidence of the Hawthorne effect. When participants are aware of their involvement in a research project, they may exhibit socially desirable behaviors in order to cultivate a positive impression. Alternatively, they may exhibit reduced expressiveness due to feelings of anxiety stemming from being under observation [99]. Furthermore, it should be noted that a potential weakness of this study is the time constraint associated with its execution. The limitation of conducting data collection techniques exclusively at a given time of day poses a potential challenge to the external validity of the study. As a result, it may be challenging to generalize the study's findings depending on the time of day that people are interviewed.

## Funding statement

This research did not receive any specific grant from funding agencies in the public, commercial, or not-for-profit sectors.

## Data availability statement

Data included in article/supp. Material/referenced in the article.

## Ethics approval and consent to participate

The facts and views in the manuscript are solely ours, and we are totally responsible for authenticity, validity, and originality. We also declare that this manuscript is our original work, and we have not copied from anywhere else. There is no plagiarism in my manuscript.

## Additional information

No additional information is available for this paper.

## CRediT authorship contribution statement

**Hasan Yıldızhan:** Supervision, Writing – review & editing. **Sahand Hosoulı:** Formal analysis, Methodology, Visualization. **Sıdıka Ece Yılmaz:** Conceptualization, Data curation, Formal analysis, Methodology, Writing – original draft. **João Gomes:** Supervision, Writing – review & editing. **Chandan Pandey:** Supervision, Writing – review & editing. **Tarik Alkharusi:** Supervision, Writing – review & editing.

## Declaration of competing interest

The authors declare that they have no known competing financial interests or personal relationships that could have appeared to influence the work reported in this paper.
